# A study of macroinvertebrate communities in Bolshiye Koty Bay of Lake Baikal using DNA metabarcoding

**DOI:** 10.18699/VJGB-23-80

**Published:** 2023-10

**Authors:** L.S . Kravtsova, T.E. Peretolchina, T.I. Triboy, I.A. Nebesnykh, A.E. Tupikin, M.R. Kabilov

**Affiliations:** Limnological Institute of the Siberian Branch of the Russian Academy of Sciences, Irkutsk, Russia; Limnological Institute of the Siberian Branch of the Russian Academy of Sciences, Irkutsk, Russia; Limnological Institute of the Siberian Branch of the Russian Academy of Sciences, Irkutsk, Russia; Limnological Institute of the Siberian Branch of the Russian Academy of Sciences, Irkutsk, Russia; Institute of Chemical Biology and Fundamental Medicine of the Siberian Branch of the Russian Academy of Sciences, Novosibirsk, Russia; Institute of Chemical Biology and Fundamental Medicine of the Siberian Branch of the Russian Academy of Sciences, Novosibirsk, Russia

**Keywords:** communities of macroinvertebrates, diversity, DNA metabarcoding, COI, high-throughput sequencing technologies, Lake Baikal, сообщества макробеспозвоночных, разнообразие, ДНК метабаркодинг, СОI, высокопроизводительное секвенирование, Байкал

## Abstract

The diversity of macroinvertebrates, the structure of their communities in Bolshiye Koty Bay (Lake Baikal) was studied by a DNA metabarcoding approach using an Illumina MiSeq system. Internal primer mlCOIintF in combination with jgHCO2198 of the Folmer fragment of the COI gene were used for macroinvertebrate metabarcoding. A total of 118009 reads of the COI gene fragment (at least 313 bp in length) were obtained. The correlation of the Spearman coefficient (S = 0.6, p<0.05) with the abundance of macroinvertebrates in the samples before DNA extraction showed that the number of reads can serve as an indirect characteristic of the abundance of a species (operational taxonomic unit, OTU). 115 OTUs belonging to the higher taxa of macroinvertebrates were identified: Porifera, 1; Platyhelminthes, 3; Annelida, 38; Arthropoda, 55; Mollusca, 18. At a high level of resolution (with homology with GenBank reference sequences ≥ 95 %, coverage ≥ 90 %), 46 taxa of macroinvertebrates comprising three communities were registered: one dominated by molluscs (Choanomphalus conf. maacki) and two dominated by chironomids (Orthocladius gregarius Linev., Sergentia baicalensis Tshern.). Communities are characterized by low species diversity according to Shannon (from 0.7 to 1.2 bits), high concentration of dominance according to Simpson (from 0.5 to 0.7) and low evenness according to Pielou (from 0.3 to 0.4). Dominants and subdominants in the communities account for 91 to 96 % of COI gene fragment reads. The spatial distribution of the dominant species identified in the communities is influenced by the geomorphological features of the bottom and the composition of sediments in the area studied. The approach proposed for studying the structure of macroinvertebrate communities based on DNA metabarcoding and next generation sequencing can be recommended for express assessment of the state of aquatic ecosystems in the monitoring.

## Abstract (ru)

Приводятся сведения о разнообразии макробеспозвоночных животных, структуре их сообществ в бухте Большие Коты оз. Байкал, полученные методом ДНК метабаркодинга на основе NGS-технологии (Illumina, MiSeq). Для ДНК метабаркодинга макробеспозвоночных был использован внутренний праймер mlCOIintF в комбинации с jgHCO2198 для амплификации фолмеровского фрагмента гена СОI. Всего получено 118 009 прочтений фрагмента гена СОI (длиной не менее 313 п. н.). Показано, что количество прочтений может служить опосредованной характеристикой обилия вида (операционной таксономической единицы – ОТЕ). Корреляция количества прочтений с численностью макробеспозвоночных в пробах до экстракции ДНК по коэффициенту Спирмена составляет 0.6 ( p < 0.05). Выявлено 115 ОТЕ, принадлежащих высшим таксонам макробеспозвоночных животных: Porifera – 1, Platyhelminthes – 3, Annelida – 38, Arthropoda – 55, Mollusca – 18. На видовом уровне (при гомологии с референсными последовательностями GenBank ≥ 95 % и покрытии не менее 90 %) зарегистрировано 46 таксонов макробеспозвоночных, формирующих три сообщества: одно – с доминированием моллюсков Choanomphalus conf. maacki и два – с доминированием хирономид Orthocladius gregarius Linev., Sergentia baicalensis Tshern. Сообщества характеризуются невысоким видовым разнообразием по Шеннону (от 0.7 до 1.2 бит), высокой концентрацией доминирования по Симпсону (от 0.5 до 0.7) и низкой выравненностью по Пиелу (от 0.3 до 0.4). На долю доминантов и субдоминантов в сообществах приходится от 91 до 96 % прочтений фрагмента гена СОI. На пространственное распределение доминирующих видов сообществ влияют геоморфологические особенности дна в исследуемом районе и состав донных отложений. Предложенный подход для изучения структуры сообществ макробеспозвоночных на основе ДНК метабаркодинга может быть рекомендован для экспресс-оценки состояния водных экосистем при мониторинге.

## Introduction

The structure of aquatic organisms communities, in particular macroinvertebrates, is one of the indicators characterizing the state of water bodies. Research in this direction is relevant in connection with global climate change and increasing anthropogenic impact on aquatic ecosystems (O’Reilly et al., 2003; Bonada et al., 2007; Burgmer et al., 2007; Moss et al., 2011; Hampton et al., 2018).

Community of organisms is a set of populations of different species coexisting in space and time (Begon et al., 1986). Their structure is formed under the influence of both abiotic environmental factors (Brauns et al., 2007; McGoff et al., 2013; Rezende et al., 2014; Worrall et al., 2014) and biotic interactions (van den Berg et al., 1997; Arbačiauskas et al., 2008; Nalepa et al., 2009). As a rule, the species diversity and abundance of organisms are the basic characteristics of the structure of communities. The study of the diversity of organisms at a high-resolution species level requires the involvement of a large number of morphologists and is associated with a laborious process of taxa identification. Currently, molecular genetics methods such as DNA metabarcoding using highthroughput sequencing technologies are used as an alternative to the classical methods of the taxonomic diversity studying of aquatic ecosystems. This method is widely used to study the diversity of both marine and freshwater fauna (Porazinska et al., 2009; Hajibabaei et al., 2011; Aylagas et al., 2014; Elbrecht et al., 2017; Haenel et al., 2017; Kuntke et al., 2020).

In the early 2000s, the mitochondrial COI gene was adopted as a standard for DNA barcoding of animal taxa (Hebert et al., 2003). Despite the universality of primers (LCO1490 and HCO2198) for the “Folmer fragment” of the mitochondrial COI gene amplification (Folmer et al., 1994), many researchers began to use different, more variable regions of it to obtain a clear phylogenetic signal. For example, for marine nematodes, region I3–M11 was used (Derycke et al., 2010). The mini-barcode of the Folmer COI fragment using a combination of primers mlCOIintF and jgHCO2198 has become popular to assess the diversity of Metazoa (Meusnier et al., 2008; Leray et al., 2013). The mini-barcode also proved to be effective in assessing the diversity of benthic invertebrates in the Listvennichny Bay of Lake Baikal (Kravtsova et al., 2021).

In the last decade, the application of environmental DNA (eDNA) approaches for rapid assessment of the biodiversity from water and sediment samples has been increased (Yu et al., 2012; Lacoursière-Roussel et al., 2018). The advantage of this method is a quick result, since no preliminary isolation of any organisms from the samples is required. However, it was less effective for studying the diversity of Metazoa in water bodies. It was shown that DNA metabarcoding of invertebrate tissues gives a more accurate estimation of the diversity of multicellular organisms (99 % of reads) than DNA from the environment (only 12 % of reads) (Gleason et al., 2021). In this study, we tried to find out the acceptability of the method of DNA metabarcoding from the tissues of organisms for assessing not only the diversity of macroinvertebrates, but also for the quantitative ratio of species that form communities.

The aim of this work is to study the features of the structural organization of macroinvertebrate communities distributed in the coastal zone of open Baikal using DNA metabarcoding.

## Materials and methods

**Fig. 1.**
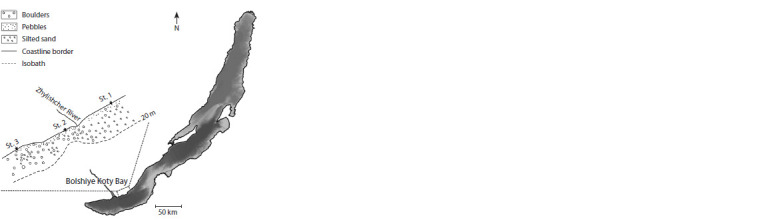
Map-scheme of sampling localities of macrozoobenthos in Bolshiye Koty Bay, Lake Baikal (July, 2019).

Quantitative samples of zoobenthos were collected in July 2019 in Bolshiye Koty Bay of Lake Baikal along the coastline over a 1 km long (Fig. 1).

Macroinvertebrates were collected from different types of bottom sediments at three stations (No. 1–3) (see Fig. 1). The first type of bottom sediment included large and small pebbles with individual boulders located in the subaqueous part of the beach (depths 0.3–0.4 m). The second type of bottom sediment was represented by boulders, unrounded rock fragments with crushed stone, and the third type was represented by silted sand. The last two types of sediments were found mainly on a shallow terrace at depths of 2–5 m (see Fig. 1). On the underwater part of the beach and on a shallow terrace samples were taken manually and with the help of divers, respectively. On each type of bottom sediment, five quantitative samples of zoobenthos were collected using a 0.1 m2 counting frame. Invertebrates from the surface of stones were brushed off into a cuvette with water, and those from silted sand were removed by flotation in a saturated sugar solution with a specific gravity of 1.12 g/L. Samples were washed through a mill sieve No. 23 and fixed with 96 % ethanol. In total, 15 quantitative samples of zoobenthos were collected and sorted under laboratory conditions using an MBS-10 microscope (at 20× magnification).

According to Elbrecht et al. (2017), preliminary sorting of organisms by size significantly improves the result of sequencing of all taxa, regardless of the biomass of the organism. Since the zoobenthos in Baikal is represented by different size groups: mega-, macro-, meioorganisms, in this work we limited ourselves to only one size group – macroinvertebrates. Their sizes in samples varied from 2 to 50 mm. All macroinvertebrates found in the sample were used for DNA extraction. First, invertebrates were soaked in distilled water (1 hour), small organisms (2–3 mm) were taken as a whole, and tissue pieces of 2–3 mm were taken from large indivi-duals (more than 5 mm) in order to level the scatter of biomass in size. Then, pieces of tissue and small organisms collected from the same type of bottom sediment were combined into one sample, placed in a porcelain cup with 2 % CTAB solution, and ground with a pestle. DNA was extracted according to a modified (chloroform was used instead of a phenolchloroform- isoamyl mixture) method of Doyle and Dickson (Doyle, Dickson, 1987). In total, three samples of genomic DNA (at least 20 ng each) of invertebrate animals were prepared for metabarcoding.

Primers mlCOIintF: GGWACWGGWTGAACWGTW TAYCCYCC (Leray et al., 2013) and jgHCO2198: TAIA CYTCIGGRTGICCRAARAAYCA (Geller et al., 2013), where “I” is inosine, were used to obtain COI gene amplicons. Amplification was performed in a volume of 20 μl containing 0.2 mM of each dNTP, 0.5 μM of each primer, 2 mM MgCl2, 5 μM SYTO9, 10 ng of DNA, 25 U/ml of Maxima Hot Start Taq DNA Polymerase (Thermo Scientific, Lithuania). Realtime PCR was performed on a CFX96 Touch Real-Time PCR system (Bio-Rad, USA) according to the program: initial denaturation at 95 °C, 4 min; 32 cycles at 95 °C – 30 s, 48 °C – 30 s and 72 °С – 30 s. The primer annealing temperature was selected using gradient PCR. PCR products were analyzed on an MCE-202 MultiNA Microchip Electrophoresis System using a DNA 12000 Reagent Kit (Shimadzu, Japan). The resulting amplicons were quantified on a Qubit 2.0 fluorimeter with a Qubit DNA High Sensitivity Assay Kits (Invitrogen, USA). NEBNext Ultra II DNA Library Prep Kit for Illumina and NEBNext Multiplex Oligos for Illumina (Dual Index Primers Set 1) (NEB, UK) were used to obtain DNA libraries from amplicons. The resulting libraries were quantified using the Kapa SYBR Fast Universal qPCR Kit (KapaBiosystems, USA). Library sequencing was performed using the MiSeq Reagent Standard Kit v3 PE300 on Miseq (Illumina, USA) at the Central Collective Use Center “Genomics” (ICBFM SB RAS).

The resulting paired reads were analyzed with UPARSE scripts (Edgar, 2013) using Usearch v11.0.667 (Edgar, 2010). Bioinformatics analysis included overlapping paired reads, filtering by quality and length, accounting for identical sequences, discarding singletons, removing chimeras, and obtaining an OTU (operational taxonomic unit). The taxonomic identification of the OTU sequences was determined using the SINTAX algorithms (Edgar, 2016) and the reference database MIDORI_UNIQUE_20180221_COI_SINTAX (Machida et al., 2017), as well as BLAST. The representativeness of OTU samples was analyzed using iNEXT 2.0.15 (Hsieh et al., 2016). The nucleotide sequences of the OTUs identified were tested for the presence of stop codons using the SeqKit v0.16.1 (https://doi.org/10.1371/journal.pone.0163962).

The Spearman correlation coefficient (S) was calculated to assess the relationship between the initial abundance of organisms of higher taxa in samples (before DNA extraction) and the number of reads of the COI gene fragment.

Communities of macroinvertebrates were identified by clustering the OTUs of the species rank and their COI reads by the Ward method; the Euclidean metric was used as a distance measure. Dominants and subdominants in the communities were determined by descending ranking of the modified density index (occurrence of OTUs in samples and their relative abundance = reads, in %) (Brotskaya, Zenkevich, 1939; Konstantinov, 1986). The communities of macroinvertebrates were named according to the dominant species with the highest density index. Species with a density index of ≥10 % were classified as subdominants. Species with a density in-dex ≤10 % were considered minor. To characterize the degree of complexity of the community structure, the Shannon diversity (H, bit), Simpson dominance (D), and Pielou evenness (e) indices were used (Odum, 1983).

The spatial distribution of dominants and subdominants of communities in Bolshiye Koty Bay was analyzed using the principal component method, the relative abundance of OTUs and the composition of bottom sediments were used as variables. The data for calculations using multivariate statistics were previously transformed by the log(X+1) function. Calculations were carried out using the statistical programming environment R 3.0.0, package vegan 2.0-7.

## Results

A total of 118,009 reads of the COI gene fragment (at least 313 bp in length) were obtained using high-throughput sequencing. Bioinformatics analysis showed that the proportion of unclassified COI sequences was less than 1 % of the total (1,157). 116,852 reads account for 115 OTUs belonging to the higher taxa of macroinvertebrates: Porifera – 1, Platyhelminthes – 3, Annelida – 38, Arthropoda – 55, Mollusca – 18.

The Spearman correlation coefficient (S) between the abundance of macroinvertebrates of higher taxa (Platyhelminthes, Hirudinea, Polychaeta, Oligochaeta, Isopoda, Amphipoda, Trichoptera, Chironomidae, Bivalvia, Gastropoda) found in samples before DNA extraction with the number of reads of the COI gene fragment is 0.6 ( p <0.05), which allows us to take the indices of the latter as equivalent to the relative abundance of organisms

Forty-six taxa of species and genus ranks with ≥98 % and ≥95 % homology with GenBank sequences, respectively, were identified with coverage of at least 90 % (Table 1). They account for 88 % of the COI gene fragment reads.

**Table 1.**
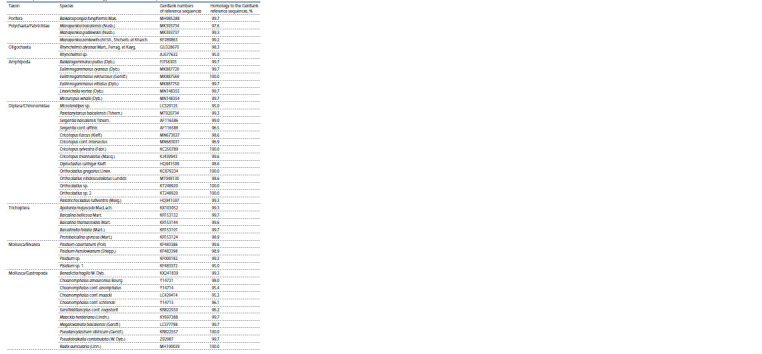
Composition of OTUs with ≥ 95% homology to GenBank reference sequences

**Fig. 2.**
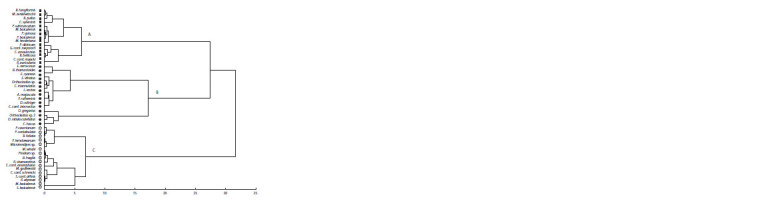
Dendrogram of OTUs of macroinvertebrates constructed by the Ward clustering method using the Euclidean metric as a measure of distance. Clusters A, B, C on the dendrogram are macroinvertebrate communities identified based on reads of the COI gene fragment.

Three communities of macroinvertebrates were identified in the region studied, one of which was dominated by the molluscs Choanomphalus conf. maacki (A) and two were chironomid-dominated by Orthocladius gregarius Linev. (B), Sergentia baicalensis Tshern. (C) (Fig. 2).

Communities are characterized by a simple structure; 15–16 species were noted in their composition (Table 2). The Shannon index is low, ranging from 0.7 to 1.2 bits. At the same time, in the communities, there is a high concentration of dominance of one species (D varies from 0.5 to 0.7) and low evenness (e varies from 0.3 to 0.4).

**Table 2.**
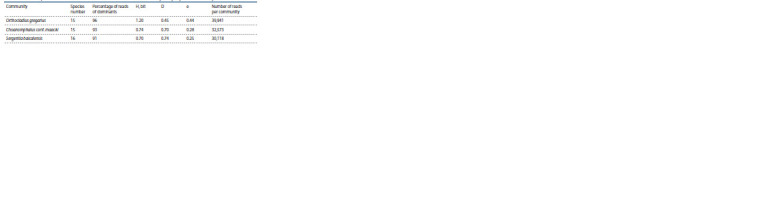
Structural parameters of macroinvertebrate communities found in Bolshiye Koty Bay, Lake Baikal (July, 2019) Notе. H – species diversity according to Shannon, D – Simpson dominance index, e – Pielou’s evenness index.

In the community C. conf. maacki among the subdominants there are molluscs Pseudancylastrum sibiricum (Gerstf.), Gerstfeldtiancylus conf. roepstorfi. The subdominants of the O. gregarius community include the caddisflies Baicalina thamastoides Mart., chironomids Orthocladius sp. 2, O. nitidoscutellatus Lundstr., and Cricotopus fuscus (Kieff.). The community dominated by S. baicalensis contains numerous polychaetes Manayunkia godlewskii (Nusb.), molluscs Choanomphalus conf. anomphalus. Dominants and subdominants in communities account for 91 to 96 % of COI gene fragment reads.

**Fig. 3.**
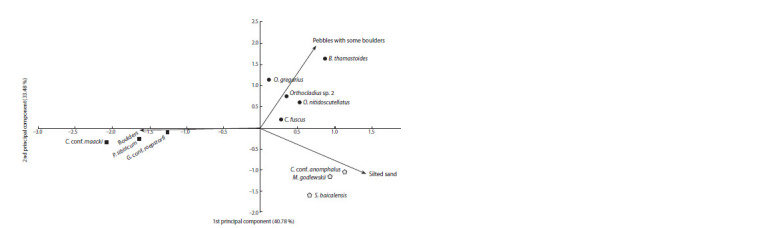
Distribution of dominant species (OTUs) of macroinvertebrate communities (A–C) in the space of the first two principal components, taking into account 74 % of the variability of the relative abundance data set.

The spatial distribution of the dominant species of macroinvertebrate communities depending on environmental factors is shown in Figure 3. The first principal component characterizes the distribution of communities depending on the composition of bottom sediments. The dominant species of macroinvertebrate communities found on fine clastic material (pebbles, silted sand) have positive loads on the first principal component, and negative loads on coarse clastic material (boulders, rock fragments). The second principal component characterizes the distribution of dominants of the studied macroinvertebrate communities depending on the bottom geomorphology. The species found in the beach area have positive loads, while those found on the shallow-water terrace have negative loads.

## Discussion

The studies of Bailey et al. (2001) indicate the effectiveness of monitoring observations of the state of water bodies using taxonomy at the genus or species level. Metabarcoding, as a modern genetic tool, is widely used for express assessment of biodiversity in ecosystems (Elbrecht, Leese, 2015). The authors of the work recommend using the presence/absence metric to characterize diversity, since the high resolution of primers makes it possible to take into account mass and minor species, but at the same time it does not make it possible to measure the absolute values of their abundance or biomass in samples. However, to study the structure of communities, indicators characterizing not only the composition of taxa, but also their quantitative ratio are needed. The number of reads, apparently, can be attributed to an indirect characteristic of species abundance (OTU). This confirms the presence of a positive correlation between the abundance of macroinvertebrates before DNA extraction and the abundance of OTUs from Lake Baikal not only in the samples collected in Bolshiye Koty Bay (S = 0.6, p < 0.05), but also in Listvennichny Bay (S = 0.5) (Kravtsova et al., 2021). In addition, a positive correlation was noted between the number of reads and the biomass of organisms collected in ponds in Germany (Elbrecht, Leese, 2015). Therefore, when studying the structure of macroinvertebrate communities, emphasis was placed on the number of reads per OTU, as well as the occurrence of OTUs in samples. To understand the extent to which the molecular genetics method using high-throughput sequencing technologies is applicable to the study of macroinvertebrate communities, the same approach was used for comparison as in ecological studies of water bodies based on classical hydrobiological methods (Brotskaya, Zenkevich, 1939; Begon et al., 1986; Konstantinov, 1986).

The fauna of macroinvertebrates on the shallow-water terrace and in the underwater part of the beach of the studied area of Lake Baikal in Bolshiye Koty Bay (excluding the population of the underwater slope and canyon) is quite diverse; in 1988, at least 177 species were recorded in its composition (Kravtsova et al., 2003). Most of the species found using DNA metabarcoding in 2019 were present here earlier, but in general, the diversity of benthic fauna (see Table 1) identified using molecular genetics methods is lower. This can be explained, on the one hand, by the smaller volume of collected quantitative samples of macrozoobenthos, and, on the other hand, by the absence in GenBank of COI sequences belonging to Baikal rich in species groups, for example, oligochaetes. In Baikal, 202 species of oligochaetes have been recorded, among them 165 are endemic (Semernoy, 2004). It is possible that the low representation of COI sequences of this group of invertebrates in GenBank can be connected with the poor knowledge of the fauna due to its high endemism. In total, Annelida accounts for about 30 % of the total number of OTUs identified by DNA metabarcoding both from Bolshiye Koty Bay and Listvennichny Bay of Lake Baikal (Kravtsova et al., 2021).

Polychaeta, which are not rich in species, are also included in Annelida, but they were not previously included in the list of taxa from Bolshiye Koty Bay (Kravtsova et al., 2003), since their morphological identification had not been carried out. However, using DNA metabarcoding, in 2019, all three polychaete species found in Baikal were recorded in the fauna with high homology (98–99 %) with sequences from GenBank (see Table 1) (Pudovkina et al., 2016): Manayunkia baicalensis (Nusb.), M. godlewskii, M. zenkewitschii Sit., Shcherb. et Kharch.

Chironomids (Diptera) are among the objects, the identification of species of which by the morphological characters of the larvae is extremely difficult and they are often determined to a group of species (gr.) or species (sp.). It is possible that O. gregarius, the dominant of one of the three communities identified in 2019, was previously listed as O. gr. thienemanni, and the subdominant O. nitidoscutellatus was in O. gr. olivaceus (Kravtsova et al., 2003). Despite these species having been present in Bolshiye Koty Bay in 1988, they did not play a community-forming role among other macroinvertebrates (Kravtsova et al., 2004). The species O. gregarius and O. nitidoscutellatus, as shown by further molecular genetic studies (Kravtsova et al., 2014), have a rather long evolutionary history and their existence in Lake Baikal is beyond doubt (Makarchenko E.A., Makarchenko M.A., 2008).

The low diversity of species rank OTUs indicates that the macroinvertebrate communities dominated by C. conf. maacki, O. gregarius, S. baicalensis in Bolshiye Koty Bay are characterized by a simple structure (see Table 2) due to the above reasons.

It is known that abiotic environmental factors play an important role in the distribution and formation of the diversity of macroinvertebrate communities (Rezende et al., 2014). The spatial distribution of dominants and subdominants of macroinvertebrate communities from Bolshiye Koty Bay is consistent with the distribution of these species in the coastal zone of Lake Baikal. So, a community dominated by C. conf. maacki is confined, as before, to the rocky bottom sediments of the shallow terrace. The community of O. gregarius is distributed on pebbles with individual boulders in the beach zone, and with the dominance of S. baicalensis, on the silty sands of a shallow-water terrace, a typical biotope for this species. In Lake Baikal, representatives of the genus Orthocladius prefer to settle in a hydrodynamically active zone of wave mixing and water flow, while Sergentia prefer to settle in conditions where sedimentation processes prevail over erosion and transfer of terrigenous material, organic matter.

The study of the structure of macroinvertebrate communities in aquatic ecosystems using DNA metabarcoding has its own characteristics, in contrast to the rapid assessment of fauna diversity (based on the presence/absence metric). First of all, it is necessary to pay attention to the degree of study of the diversity of the bottom fauna of the reservoir, the size groups of its representatives. Of no small importance for the assessment of diversity is the presence of sequences of the studied gene fragment in the GenBank database. To obtain an adequate characterization of the relative abundance (reads of the COI gene fragment) of organisms in the community, quantitative sampling of macrozoobenthos should be carried out from a certain area, taking into account the biotopic heterogeneity of the bottom. In order to avoid the influence of body size of organisms on the number of reads per OTU, when preparing samples for DNA extraction, it is necessary to select tissue pieces of the same size from all individuals found in quantitative samples. This makes it possible to obtain an integral characteristic of the relative abundance of an organism (number of reads), taking into account its biomass, leveled by scatter (due to body size), as well as abundance. Since macroinvertebrates make up one third of the representatives of the unique Baikal fauna (2,565 species and subspecies (Timoshkin, 1997)), a more complete database of COI reference sequences is required for an objective assessment of α-diversity.

## Conclusion

DNA metabarcoding using a primer (mlCOIintF) in combination with jgHCO2198 to amplify the Folmer COI gene fragment showed its effectiveness in studies of the diversity and structure of Baikal macroinvertebrate communities. The fauna of Bolshiye Koty Bay contains typical representatives of most groups of macroinvertebrates inhabiting the coastal zone of Lake Baikal. It is shown that the number of reads, as a characteristic of the relative abundance of taxa, can be used to study the features of the structural organization of macroinvertebrate communities. In general, the proposed approach is quite acceptable for assessing the stability of macroinvertebrate communities in the temporal aspect in the monitoring of aquatic ecosystems.

## Conflict of interest

The authors declare no conflict of interest.
